# Urine-derived induced pluripotent stem cells for non-invasive diagnosis of bladder cancer

**DOI:** 10.7150/jca.138160

**Published:** 2026-09-11

**Authors:** Yuan Zhu, Jia Shang, Xin Zhang, Sheng Tu, Gang Li, Shenjuan Li, Hongwei Peng, Haoli Ma, Fangjin Chen, Wan Xiang, Cong Zou, Yu Xiao, Zilin Xu, Kaiyu Qian, Gang Wang

**Affiliations:** 1 Department of Biological Repositories, Zhongnan Hospital of Wuhan University, Wuhan, Hubei, 430071, China.; 2 Human Genetic Resources Preservation Center, Zhongnan Hospital of Wuhan University, Wuhan, 430071, China.; 3 Laboratory of Precision Medicine, Zhongnan Hospital of Wuhan University, Wuhan, Hubei, 430071, China.; 4 Department of Urology, Hubei Key Laboratory of Urological Diseases, Zhongnan Hospital of Wuhan University, Wuhan, China.; 5 Brain Glioma Center & Department of Neurosurgery, Zhongnan Hospital of Wuhan University, Wuhan, 430071, China.; 6 Center for Quantitative Biology, School of Life Sciences, Peking University, Beijing, 100871, China.; 7 Department of Laboratory Medicine, Zhongnan Hospital of Wuhan University, Wuhan, 430071, China.

**Keywords:** urine-derived iPSCs, bladder cancer, non-invasive diagnosis, cellular reprogramming, urothelial cells, disease modeling, liquid biopsy, precision oncology

## Abstract

Bladder cancer (BLCA) remains one of the most prevalent and costly malignancies worldwide, largely owing to its high recurrence rate and the invasive nature of conventional surveillance modalities. Induced pluripotent stem cell (iPSC) technology offers a new framework for disease modeling and, potentially, precision diagnostics. Urine provides a non-invasive, renewable source of exfoliated urothelial and renal epithelial cells amenable to direct reprogramming. Here, we review the biological rationale, reprogramming methodology, and diagnostic potential of urine-derived iPSCs (UDiPSCs) in the context of BLCA detection and monitoring. We further discuss the genomic fidelity of UDiPSCs, the extent to which they may retain patient-relevant oncogenic features, their integration with multi-omics platforms, and the translational challenges that must be resolved before clinical deployment. Because reprogramming resets the somatic epigenome and transcriptome, we propose a tiered strategy in which methylation is assessed in parental urinary cells, genomic variants in reprogrammed clones, and transcriptional phenotypes in differentiated derivatives. Compared with urinary tumor DNA methylation panels and urine-derived tumor organoids, which currently exceed cell-based reprogramming approaches in accuracy, cost-effectiveness, and turnaround, UDiPSCs offer a distinct niche: interrogating rare, non-tumorigenic, field-altered urothelial cells that are diluted in bulk urinary DNA and do not form organoids. Collectively, UDiPSC-based platforms provide a plausible route toward non-invasive, personalized BLCA biomarkers, although clinical utility remains to be established through prospective validation.

## 1. Introduction

### 1.1 The unmet diagnostic need in bladder cancer

The central unmet need in bladder cancer (BLCA) is not detection per se, but a non-invasive modality capable of replacing repeated cystoscopy without sacrificing diagnostic accuracy. Cystoscopy, the reference standard, is invasive, operator-dependent, and poorly suited to the frequent repetition that surveillance demands 1. Its principal non-invasive adjunct, urine cytology, is highly specific but insensitive precisely to the lesions that dominate routine surveillance, detecting fewer than 16% of low-grade tumors 2.

The consequences of this gap are magnified by the natural history of the disease. More than 75% of cases present as non-muscle-invasive bladder cancer (NMIBC), a category defined clinically by persistently high recurrence and a consequent requirement for lifelong monitoring 3. Surveillance intensity, rather than incidence alone, is what makes BLCA one of the most expensive malignancies to manage on a per-patient basis 4. The scale of the affected population is substantial: in 2022, an estimated 614,298 new cases and 220,596 deaths were recorded worldwide, with marked sex- and geography-related variation in incidence 5, 6. A test that reduced even a fraction of surveillance cystoscopies would therefore have disproportionate clinical and economic effect.

This mismatch, between the density of surveillance required and the performance of the non-invasive tools available to deliver it, defines the problem this review addresses. Against this backdrop, we examine whether urine-derived induced pluripotent stem cell (UDiPSC) approaches can contribute a distinct and defensible advantage, and, equally importantly, where they cannot.

### 1.2 The promise of UDiPSC-based diagnostics

Since the landmark demonstration by Takahashi and Yamanaka in 2006 that somatic cells could be reprogrammed to a pluripotent state through forced expression of defined transcription factors (OCT4, SOX2, KLF4, c-MYC; OSKM), iPSC technology has undergone extraordinary refinement 7. Integration-free episomal vectors enabled footprint-free reprogramming and reduced the confounding effects of vector integration, which is advantageous for diagnostically oriented applications 8. Integration-free iPSC derivation preserves donor-specific genetic background more effectively than integrating approaches; however, reprogramming and subsequent expansion can introduce or select additional genomic variation. Accordingly, preservation of pre-existing somatic variants and copy-number states should be established empirically by paired analysis of parental cells and derived iPSC clones for each intended diagnostic application 8-11.

In oncology, patient-derived iPSC workflows can provide renewable material for downstream molecular and functional studies 12. However, whether reprogramming of urine-derived cells consistently enriches for diagnostically relevant tumor-associated clones in BLCA remains unproven and should be evaluated in paired parental-cell/iPSC datasets with explicit assessment of clonal selection bias 11, 13, 14. When the source material is urine, this framework achieves complete non-invasiveness, a particularly compelling advantage for a disease in which exfoliated cellular material can be recovered from urine and that often requires repeated long-term surveillance 3, 15. Throughout this mini-review, UDiPSC-based diagnostics are discussed as a hypothesis-generating and potentially enabling framework rather than as a clinically validated testing strategy (Figure 1).

## 2. Urine as a biospecimen: biological composition and cellular yield

### 2.1 Cellular constituents of voided urine

Voided urine carries cells shed from the full length of the urinary tract: renal tubular epithelium, urothelium of the bladder and upper tract, and rare progenitor-like populations 16, 17. The predominant nucleated cells recovered from voided urine are urothelial and renal epithelial cells, and typical midstream samples from healthy donors can yield on the order of 10^3^-10^4^ viable cells per 100 mL, although cell number and composition vary substantially across donors and clinical contexts 13, 15, 16. For patients with BLCA the corresponding numbers are unknown. No study has reported cell recovery or viability from bladder cancer urine. Gross hematuria, active inflammation, and recent intravesical BCG or chemotherapy would each be expected to change what is recoverable, but the direction and magnitude of these effects have not been measured. We flag them here as an untested assumption rather than an established confounder.

Zhou and colleagues reported in 2011 that cells pelleted from urine by ordinary centrifugation could be expanded in defined medium and reprogrammed to bona fide iPSCs, initially with retroviral factor delivery, and set the method out in protocol form the following year 13, 15. Reported reprogramming efficiency reached 4% 13, 15. Later work replaced integrating vectors with episomal and other non-integrative systems and tightened the isolation and culture steps. That is what made the approach reproducible outside the originating laboratory 18-20.

The populations recoverable from urine are not interchangeable, and conflating them has consequences for expansion potential, senescence kinetics, and reprogramming efficiency. Urine-derived stem cells (USCs) are a rare subset carrying MSC surface markers (CD73, CD90, CD105) alongside pericyte-associated antigens, and they expand and differentiate extensively 17. Bulk urine-derived cells, the starting material in the Zhou protocol, are something else: a mixed population of shed urothelial and renal tubular epithelial cells without MSC-like self-renewal, arresting after a limited number of passages 13, 15. Urothelial cells proper are the stratified epithelium lining the tract, identifiable by uroplakin and cytokeratin expression 16, 17. Nearly all the reprogramming literature discussed in this review, including the foundational reports, used bulk urine epithelial cells rather than prospectively purified USCs 13, 15. Whether efficiency and clonal behavior translate to purified USCs has not been tested directly and cannot be assumed 17, 18.

Anatomy makes this cell source more than a convenience. Field cancerization in the bladder holds that genetically altered urothelium can be histologically normal, and can sit adjacent to a visible tumor or precede one 21. TERT promoter mutations, and in some series other BLCA driver events, have indeed been detected in normal-appearing urothelium 22. If shed cells sample that altered field, urine-derived cells become a candidate sentinel population for surveillance 6, 22. That inference is at present a rationale for testing the idea, not evidence that it works.

### 2.2 Advantages of urine over alternative cell sources

Compared with other commonly used somatic cell sources for reprogramming, urine-derived cells offer several practical and biological advantages that are particularly relevant to BLCA-oriented applications 15, 23 (Table 1).

Voided urine can be collected serially in a non-invasive manner with minimal procedural burden, making it attractive for repeated sampling in surveillance-oriented settings 15, 24. In addition, urine-derived cells include populations shed from the urinary tract, such as urothelial and renal epithelial cells, and therefore offer closer anatomical relevance to BLCA-oriented applications than peripheral biospecimens 16, 17. Bladder field cancerization implies that shed urine cells carry diagnostically informative variants even when cytologically benign, although this concept remains to be validated in dedicated urine-cell diagnostic workflows 21, 22. Compared with tissue procurement workflows that require enzymatic dissociation, urine collection avoids additional tissue-processing steps and therefore reduces some sources of handling-related cellular stress and dissociation-associated transcriptional artifacts before downstream analysis 15, 25. Finally, non-invasive collection is likely to improve acceptability for repeated sampling, although patients generally require urinary tests to approach cystoscopic accuracy before accepting them as a replacement in surveillance 24.

## 3. Reprogramming urine-derived cells to iPSCs: technical considerations

### 3.1 Cell isolation and expansion protocols

Optimal recovery is generally achieved from freshly collected midstream urine (commonly 100-200 mL) processed as soon as possible after voiding, ideally within a few hours to maximize cell viability 13, 16, 18. Following low-speed centrifugation (400 × g, 10 minutes), pelleted cells are resuspended and plated on gelatin-coated surfaces in renal epithelial growth medium supplemented with epidermal growth factor (EGF), hydrocortisone, and B-27 supplement 13, 18. Under these conditions, adherent colonies often become apparent within several days, and expandable urine-derived cell cultures can usually be maintained for multiple passages before growth arrest becomes evident 13, 16, 18.

For BLCA-oriented workflows, the pre-reprogramming expansion phase could be used for preliminary assessment of cellular atypia or canonical urothelial aneuploidy, for example by cytology or UroVysion FISH 26, 27. The incorporation of broader copy-number profiling approaches, such as shallow whole-genome sequencing, should currently be regarded as a proposed extension that requires dedicated validation in this specific pre-reprogramming setting 28.

### 3.2 Reprogramming strategies

Several reprogramming platforms have been explored for urine-derived cells, including integrating viral, episomal, and RNA-based approaches, each with distinct tradeoffs for diagnostic-oriented applications 8, 15, 20, 29. Integrating viral vectors were historically effective but are disadvantaged by insertional mutagenesis risk, which complicates downstream genomic interpretation in diagnostic settings 15. Episomal plasmid reprogramming is currently the most widely used practical non-integrating strategy for urine-derived iPSC generation in diagnostic-oriented workflows 8, 13, 20. A footprint-free delivery strategy is advantageous for downstream genomic interrogation, but retention of the parental somatic mutational landscape still requires empirical verification in each diagnostic application 8-11. In practice, urine-derived cells are reprogrammed using non-integrating episomal delivery of OSKM, with or without LIN28, and colonies typically emerge within approximately 3-4 weeks under optimized conditions 19, 20 (Figure 2). Modified mRNA provides transient, non-integrating delivery and requires repeated transfections; it has also been successfully applied to urine-derived cells 29, 30. Protein-based reprogramming has been demonstrated in principle, but it remains inefficient and has not been established for urine-derived cells at diagnostic scale 31.

### 3.3 Validation of pluripotency

Generated UDiPSCs should undergo baseline pluripotency characterization before downstream diagnostic-oriented use, including ESC-like colony morphology, expression of canonical pluripotency markers, platform-appropriate confirmation of exogenous vector/transgene loss or silencing, *in vitro* tri-lineage differentiation, and genomic integrity assessment 13, 15, 32. These criteria help reduce the risk of incomplete reprogramming, residual exogenous factor persistence, or acquired genomic instability, all of which could confound downstream molecular analyses 9, 33, 34.

## 4. Genomic fidelity and oncogenic landscape preservation in UDiPSCs

### 4.1 Somatic mutation retention during reprogramming

A central premise of the UDiPSC diagnostic paradigm is that diagnostically relevant somatic variants present in parental urine-derived cells may be retained in derived iPSC clones, although the extent and fidelity of retention require empirical verification 9-11, 35. For diagnostic applications, concordance between parental urine-derived cells and derived iPSC clones should be verified by paired sequencing, because reprogramming can retain pre-existing variants but can also introduce or enrich additional variants during derivation and culture 9-11, 35, 36. If a mutation-carrying urine-derived cell is successfully reprogrammed, the resulting iPSC clone would be expected to show relative enrichment of that variant compared with the corresponding bulk urine-cell population, although the observed variant allele fraction (VAF) would still depend on zygosity and copy-number state 11, 28, 35 (Figure 3).

This potential clonal enrichment effect is conceptually attractive in BLCA, where FGFR3 alterations are common in low-grade NMIBC, TERT promoter mutations are among the most frequent somatic events in BLCA overall, and TP53 mutations are common in MIBC 37-39. However, the extent to which UDiPSC derivation improves real-world diagnostic sensitivity over direct urine-cell or urinary cfDNA analysis remains to be established in prospective comparative studies 40.

### 4.2 Epigenetic reprogramming and diagnostic implications

Reprogramming to pluripotency entails extensive epigenetic remodeling, including widespread resetting of DNA methylation and re-establishment of pluripotency-associated chromatin states 41. The overwhelming majority of somatic, lineage- and disease-associated methylation marks are erased as cells acquire the hypomethylated promoter state characteristic of pluripotency, and diagnostically informative urinary markers, including methylated TWIST1, NID2, and BCL2 genes 42-44, cannot be assumed to survive this transition. This is not a limitation to be mitigated but a defining property of the process, and it dictates the architecture of any methylation-based readout: the epigenetic signal of interest resides in the parental urinary cells, not in the undifferentiated UDiPSC clone.

Although residual epigenetic memory has been reported in some iPSC systems, its extent is variable and typically diminishes with continued culture 45. It is therefore best treated as an empirical question, to be resolved by paired analysis of parental cells and derived clones, rather than as a dependable diagnostic substrate. Treating the undifferentiated clone as a direct readout of tumor methylation is inconsistent with what reprogramming does to the somatic methylome, and we do not propose it.

The design instead assigns each analyte to the stage at which it remains interpretable. (i) Pre-reprogramming: Methylation profiling is performed on the freshly isolated urinary cell fraction, before any factor delivery, while somatic marks are still intact. This is the only point in the workflow at which the established utDNA methylation signal can be read directly. (ii) Post-reprogramming: UDiPSC clones are interrogated for genomic variants rather than for methylation, exploiting their clonal purity to resolve low-frequency drivers otherwise diluted in bulk urinary DNA. (iii) Post-differentiation: transcriptional and functional readouts are obtained from differentiated urothelial derivatives and interpreted as differentiation-associated lineage programs rather than as recovered tumor signatures. Each layer therefore contributes something the others cannot, without any layer being asked to report on a molecular feature that reprogramming has already erased or rewritten.

## 5. Diagnostic applications of UDiPSCs in BLCA

### 5.1 Genomic profiling framework for early detection

A central proposed diagnostic application of UDiPSCs is their use as a renewable platform for downstream somatic mutation profiling, provided that genomic integrity and concordance with parental cells are empirically verified 9, 11, 32, 35. The proposed workflow (Figure 3) for NMIBC diagnostics using UDiPSCs begins with non-invasive midstream collection, centrifugation, and culture expansion under defined epithelial/renal epithelial conditions 13, 16, 19. During the pre-reprogramming phase, the expanded cells can be assessed by cytology, UroVysion FISH, and DNA methylation, with broader approaches such as shallow whole-genome sequencing (sWGS) remaining investigational in this setting 26-28. Cells are then reprogrammed using non-integrating episomal delivery of OSKM, with or without LIN28, and colonies typically emerge within approximately 3-4 weeks under optimized conditions 8, 19, 20. Pluripotency quality control includes marker expression, genomic integrity assessment, tri-lineage differentiation, and platform-appropriate confirmation of vector/transgene loss or silencing 13, 15, 32. Finally, multi-omics profiling, comprising targeted or whole-exome sequencing and RNA-seq, provides a proposed integrative analytical framework 39, 46, 47.

Following this workflow, potential downstream outputs may include: (i) Exploratory alignment with established molecular classification frameworks should be restricted to appropriately differentiated, disease-relevant models that retain tumor-relevant transcriptional features; in practice, the strongest current reference frameworks are those developed for muscle-invasive bladder cancer (MIBC) rather than for the full spectrum of BLCA states 39, 46, 47. (ii) Variant calls should be interpreted against established BLCA driver landscapes. Any statement about therapeutic actionability or risk stratification stays investigational until it is supported by disease-context-specific evidence and prospective validation 37-39, 46, 48. We treat this as a reporting rule for the framework, not a caveat to be relaxed later. (iii) The tiered architecture assigns three analytes to three stages: methylation screening on the urinary cell fraction before reprogramming, while somatic marks are intact; genomic variant detection in UDiPSC clones after reprogramming, where clonal descent from a single founder raises the sensitivity for low frequency events; transcriptional and functional readouts from differentiated urothelial derivatives.

The design resembles integrated molecular characterization as practiced in TCGA-scale efforts, but only in structure. TCGA profiled the tumor tissue itself. Here every layer reports on a surrogate that has passed through reprogramming, differentiation, or both, so concordance with tumor-derived data is an open empirical question rather than an inherited property. Clinical validity of UDiPSC-derived readouts remains unestablished in prospective studies 39, 46.

### 5.2 Disease modeling for functional diagnostics

UDiPSCs can be differentiated toward urothelial lineages *in vitro*, which extends their use beyond static genomic profiling to phenotypic assays 49, 50. Published hPSC/hiPSC urothelial differentiation protocols commonly follow an endodermal developmental trajectory, for example through definitive endoderm and caudal hindgut-like intermediates, with subsequent induction of urothelial maturation by retinoid-based conditions and terminal differentiation cues. Optimized cultures yield cells expressing uroplakins and CK20 49, 50, and terminal identity is confirmed by tight-junction formation (e.g., ZO-1, selected claudins) and functional barrier assays (e.g., TEER or permeability tests) 49, which together indicate acquisition of urothelial barrier properties.

UDiPSC-derived urothelial differentiation systems may serve as a post-detection functional validation platform for studying how defined genetic alterations influence urothelial phenotypes 49, 50. In principle, UDiPSC clones that harbor candidate driver mutations such as activating FGFR3 mutations or TERT promoter mutations could be differentiated into urothelial cells to assess the effects of these mutations on proliferation, differentiation, barrier function, and drug sensitivity. Because UDiPSC lines can be expanded indefinitely and genetically manipulated, they offer a renewable substrate for systematic genotype-phenotype studies. At present, direct evidence that UDiPSC-derived bladder models reproducibly recapitulate patient-specific tumor behavior and predict therapeutic response remains limited; the strongest functional precision-oncology data in BLCA currently come from patient-derived tumor organoids and related epithelial organoid systems rather than from UDiPSC-derived organoids 51. Therefore, UDiPSC-based disease modeling should be positioned as a complementary, hypothesis-testing platform rather than as a replacement for organoid-based functional assays or clinical validation.

The most direct comparator for a urine-derived cell-based platform is urine-derived bladder cancer organoids (urinoids), which have been established from voided or catheterized urine in 12 of 22 patients (55%), retain molecular subtype, and reproduce the drug responses of matched tissue-derived organoids; in a patient analyzed by whole-genome sequencing, single-nucleotide polymorphism and indel concordance with matched tumor tissue reached 92.56% and 91.54%, respectively 52. Comparable feasibility has been reported independently, with organoid establishment and initial expansion from urine achieved in 83% of 29 patients 53. Urinoids preserve the somatic epigenome and transcriptome, require no reprogramming, and are available in weeks. By contrast, reprogramming alone requires 3-4 weeks, and the complete UDiPSC pipeline, including pluripotency quality control, clonal expansion, and directed differentiation, cumulatively requires months. On every axis relevant to characterising an established tumor, urinoids currently dominate UDiPSC-based approaches.

The distinction that leaves conceptual space for UDiPSCs is therefore narrow and must be stated precisely. Urinoid outgrowth requires cells competent to form tumor organoids, and such a platform is by construction a tumor-cell assay. Clonal reprogramming, by contrast, is agnostic to tumorigenic potential: it can in principle capture rare, non-tumorigenic, cytologically benign field-altered urothelial cells carrying early driver events. If UDiPSC-based genomics has a defensible niche, it lies here, in interrogating the pre-malignant field rather than the established tumor, and not in competing with urinoids or tumoroids for tumor characterisation.

### 5.3 Integration with liquid biopsy platforms

UDiPSC-based diagnostics are not best viewed as a standalone replacement for established liquid biopsy modalities, but rather as a potentially complementary platform 40, 54, 55. Urinary cell-free DNA analysis and urine-derived extracellular vesicle RNA/miRNA assays capture distinct fractions of tumor-derived molecular information, although the maturity of evidence differs across analyte classes 40, 56. In BLCA, urinary cell-free DNA and urinary exosomal RNA/miRNA assays may provide complementary molecular readouts 40, 56, 57. Integrating such analytes with cell-based genomic analysis represents a plausible future composite diagnostic strategy, but any combined model would require prospective head-to-head validation rather than extrapolation from single-modality studies 40, 54, 56, 57. Blood-based multi-analyte cancer detection studies in other malignancies suggest that combining analyte classes can improve detection performance in principle, but direct translational extrapolation to BLCA remains unproven 58.

## 6. Comparative diagnostic performance: UDiPSCs versus established diagnostic modalities and selected non-invasive tests

A particularly important comparator for any emerging urine-based BLCA assay is urinary tumor DNA (utDNA) methylation profiling, which has matured rapidly in recent years. Chen and colleagues developed an efficient method called urine tumor DNA methylation MassARRAY (utMeMA) that, in a multicenter prospective validation cohort, achieved 90.0% sensitivity and 83.1% specificity for BLCA detection; critically, its sensitivity for early-stage (Ta, low-grade) tumors was 64.5%, substantially exceeding that of urine cytology (11.8%) and UroVysion FISH (15.8%) in the same study 59. Xiao and colleagues developed a panel based on utDNA methylation, enabling pre-surgery utDNA methylation testing that outperformed FISH in detecting high-grade BLCA with 100% sensitivity and low-grade BLCA with 62% sensitivity, both at 100% specificity 44. In a subsequent large prospective multicenter study of 1,099 patients with hematuria, Jeong and colleagues reported that a urinary PENK methylation test reached 89.2% sensitivity and 87.8% specificity for high-grade or invasive BLCA, with a negative predictive value of 97.6%; its sensitivity exceeded that of both the NMP22 test and cytology, although its specificity was lower than that of either comparator and the positive predictive value for high-grade or invasive disease was 61.3% 60. This performance profile is informative for positioning rather than merely for benchmarking. The principal limitation of utDNA methylation panels is not sensitivity but false positivity: a high negative predictive value permits confident exclusion, whereas a positive predictive value near 60% implies that a substantial minority of test-positive patients undergo cystoscopy unnecessarily 60. A slower but analytically orthogonal, clone-resolved assay is therefore better conceived as a candidate confirmatory test applied to methylation-positive, cystoscopy-eligible patients than as a competing primary screen. Whether UDiPSC-based genomics can occupy that position depends on demonstrating high specificity at clone level, which remains untested.

Because prospective diagnostic-accuracy data for UDiPSC-based BLCA testing are not yet available, comparison with established diagnostic modalities and non-invasive assays is currently best framed qualitatively 40, 55, 61. Table 2 therefore summarizes literature-reported characteristics of existing tests and the current evidence status of the UDiPSC approach 55, 62. Thus, comparisons between UDiPSC-based workflows and established assays should therefore be interpreted as comparisons of conceptual scope and evidence maturity rather than of validated diagnostic performance.

At present, any potential advantage of UDiPSC-based genomics for low-grade tumor detection should be regarded as a hypothesis derived from clonal-purity considerations rather than as a validated performance characteristic 11, 35, 40. Determining whether this strategy improves sensitivity over direct urine-cell or urinary cfDNA analysis will require prospective head-to-head studies 40, 55.

## 7. Technical and translational challenges

### 7.1 Reprogramming efficiency and turnaround time

Typical non-integrative urine-cell reprogramming workflows often require approximately 3-4 weeks, which remains a practical limitation for diagnostic applications requiring rapid turnaround 13, 19, 29. Acceleration strategies explored in the broader iPSC reprogramming literature include small-molecule enhancement of reprogramming kinetics (for example valproic acid, CHIR99021, ALK5/TGF-β pathway inhibitors, and tranylcypromine) and direct transcription factor protein delivery 31, 63, 64. In some optimized research systems, including selected urine-derived-cell workflows, reprogramming kinetics can be shortened to approximately 10-14 days; however, further refinement is required before such accelerated protocols meet the reproducibility and robustness standards expected for clinical-grade deployment 19, 29, 32, 64.

### 7.2 Genomic instability during reprogramming

iPSC generation is associated with a low but non-trivial burden of acquired or enriched genomic alterations, including CNVs and SNVs, arising from a combination of parental-cell heterogeneity, reprogramming-associated stress, and subsequent culture selection 9-11, 35. Distinguishing bona fide diagnostic somatic mutations inherited from the parental urine cell from de novo or selectively enriched variants requires paired sequencing of pre-reprogramming urine-derived cells and derived iPSC clones 33-36. Deriving several independent clones from the same parental cell population, and interpreting variants by consensus across those clones, reduces the risk of reporting clone-specific artifacts as clinically relevant mutations 11, 33, 35.

### 7.3 Standardization and regulatory considerations

Clinical translation will require work on four fronts: (i) Urine collection, storage, and processing need harmonized protocols across sites, with defined handling parameters 65; (ii) Workflows should be validated for the specific analytical use rather than for research-grade characterization 32, 66, 67; (iii) Variant-calling thresholds, filtering rules, and reporting frameworks should be locked before validation, not tuned afterward 68; and (iv) The strategy depends on how the assay is eventually classified in each jurisdiction 66, 69. Because the regulatory status of such workflows may differ across jurisdictions and is not yet well defined for this type of cell-derived diagnostic concept, these considerations should be viewed as illustrative rather than prescriptive. Early engagement with regulatory authorities remains advisable for defining analytical validation, quality control, and release criteria 66, 69.

### 7.4 Cost and scalability

UDiPSC derivation combined with downstream genomic profiling is likely to be resource-intensive relative to currently used urinary biomarker assays, although direct comparative cost analyses in BLCA-specific diagnostic workflows are not yet available 70, 71. Improving economic viability will likely depend on workflow automation, increased sample multiplexing for parallel processing, and, where clinically appropriate, consideration of targeted panel-based genomic assays rather than broader whole-exome approaches in initial deployment 70, 71. Health economic modeling will be needed to define the healthcare-system value proposition of UDiPSC-based testing and to inform reimbursement discussions. Such models should consider scenarios including avoided cystoscopies, altered surveillance intensity among correctly risk-stratified patients, and potential savings associated with earlier detection 72, 73.

## 8. Emerging technologies and future directions

### 8.1 Artificial intelligence for integrated diagnostic classification of UDiPSC-derived multi-omics data

In principle, machine-learning models trained on integrated UDiPSC-derived multi-omics data could support composite diagnostic classifiers for NMIBC/MIBC detection and surveillance, drawing on precedent from multi-omics machine-learning studies in BLCA and oncology more broadly 74. Within the UDiPSC-BLCA pipeline, such models could integrate genomic variants from UDiPSC clones, methylation signatures obtained from the parental urinary-cell fraction, and transcriptional readouts from differentiated derivatives, each analyzed at the tier at which it is biologically valid. Deep-learning analysis of bladder organoid images has been shown to improve segmentation and quantitative phenotyping in preclinical settings, and similar approaches could, in principle, be adapted to UDiPSC-derived urothelial models for high-throughput morphological readouts linked to disease state 75. Explainability frameworks, including feature-attribution and concept-based methods, are likely to be important if AI-derived outputs are to be interpretable and clinically reviewable in relation to established BLCA biology 76. In addition, federated learning provides one strategy for training multicenter models without the need to transfer raw patient-level data; if the UDiPSC cohorts at each research institution remain small, this method may be particularly suitable for integrating multicenter data 77.

### 8.2 CRISPR-based functional screening for variant annotation and target discovery in BLCA diagnostics

CRISPR-based perturbation in differentiated iPSC or organoid systems may, in principle, support functional interrogation of selected variants and discovery of genotype-specific dependencies 78-80. In the context of the UDiPSC-BLCA diagnostic workflow, this approach would most logically be positioned after the genomic variant-detection arm, where candidate driver mutations or variants of uncertain significance identified in reprogrammed clones could be functionally annotated to strengthen diagnostic or prognostic interpretation. However, this should presently be framed as a methodological opportunity rather than an established UDiPSC-BLCA workflow, because the strongest supporting evidence comes from organoid CRISPR platforms in other epithelial systems 78-80.

### 8.3 Direct conversion as a reprogramming-independent rapid pre-screening alternative in BLCA diagnostics

Direct conversion of urine-derived cells to neural and neural stem cell-like fates has been reported, and in some experimental settings this may provide a faster alternative to pluripotency-based workflows 13, 81, 82. For BLCA diagnostics, however, its utility remains speculative, and it should not be presented as a validated substitute for UDiPSC-based approaches. Direct conversion skips the clonal amplification step that iPSC derivation provides, and that step is what supports detection of low-frequency variants. What it offers in exchange is time. On that basis it is worth exploring as a rapid exploratory assay placed upstream of full UDiPSC derivation, with the two approaches treated as complementary rather than interchangeable. Neither the assay nor the tiered arrangement has been tested in BLCA diagnostics 81, 82.

## 9. Conclusion

Urine-derived iPSC technology draws on stem cell biology, cancer genomics, and non-invasive diagnostics. Its appeal rests on two features. Urinary cell populations originate in the tissue of interest, and clonal derivation permits downstream analyses that bulk urine samples do not support. Together, these give a framework for future bladder cancer biomarker development 6, 15, 83. Clinical relevance is still unestablished. Translation will require validation of analytical validity, reproducibility, and quality control, and it will also require prospective evaluation of turnaround time and of diagnostic performance measured directly against existing methods. The relevant comparator is utDNA methylation testing, which now sets the non-invasive benchmark 32, 40, 55, 59, 60, 66, 67, 69.

It should be clearly acknowledged that there is currently limited direct evidence that UDiPSC-derived bladder models reproducibly recapitulate patient-specific tumor behavior and predict therapeutic response, and that the central proposition of this review, that clonal derivation enriches low-frequency driver signals beyond what bulk urinary DNA affords, remains a hypothesis awaiting prospective, head-to-head evidence. Based on current evidence, established utDNA methylation assays outperform cell-based workflows in accuracy, cost-effectiveness, and clinical readiness. The value of the UDiPSC approach, if any, will be decided not by its technical elegance but by whether controlled studies demonstrate a specific advantage that no simpler method can provide. We therefore frame this pipeline as a research agenda, not a clinical proposition.

The possibility that clonal derivation may improve detection of low-frequency driver alterations is one of the platform's most distinctive hypotheses, particularly for low-grade NMIBC in which existing non-invasive modalities remain inadequate 11, 37, 38, 55. Testing this proposition will require prospective multicenter studies with prespecified performance thresholds against concurrent cystoscopic and histopathological reference standards 3, 84.

This review's contribution is not an endorsement but a specification. We have identified the specific scenario in which clonal reprogramming may be most informative, if validated: the interrogation of rare, non-tumorigenic, field-altered urothelial cells that are otherwise diluted or overlooked in bulk urinary DNA analysis. We have also specified, within this proposed application, the analytical tier at which each molecular layer remains biologically valid. Framed in this way, the value of the platform becomes an empirical question that can be answered through controlled studies rather than an open-ended promise, allowing readers to judge whether the required investment is warranted.

## Figures and Tables

**Figure 1:**
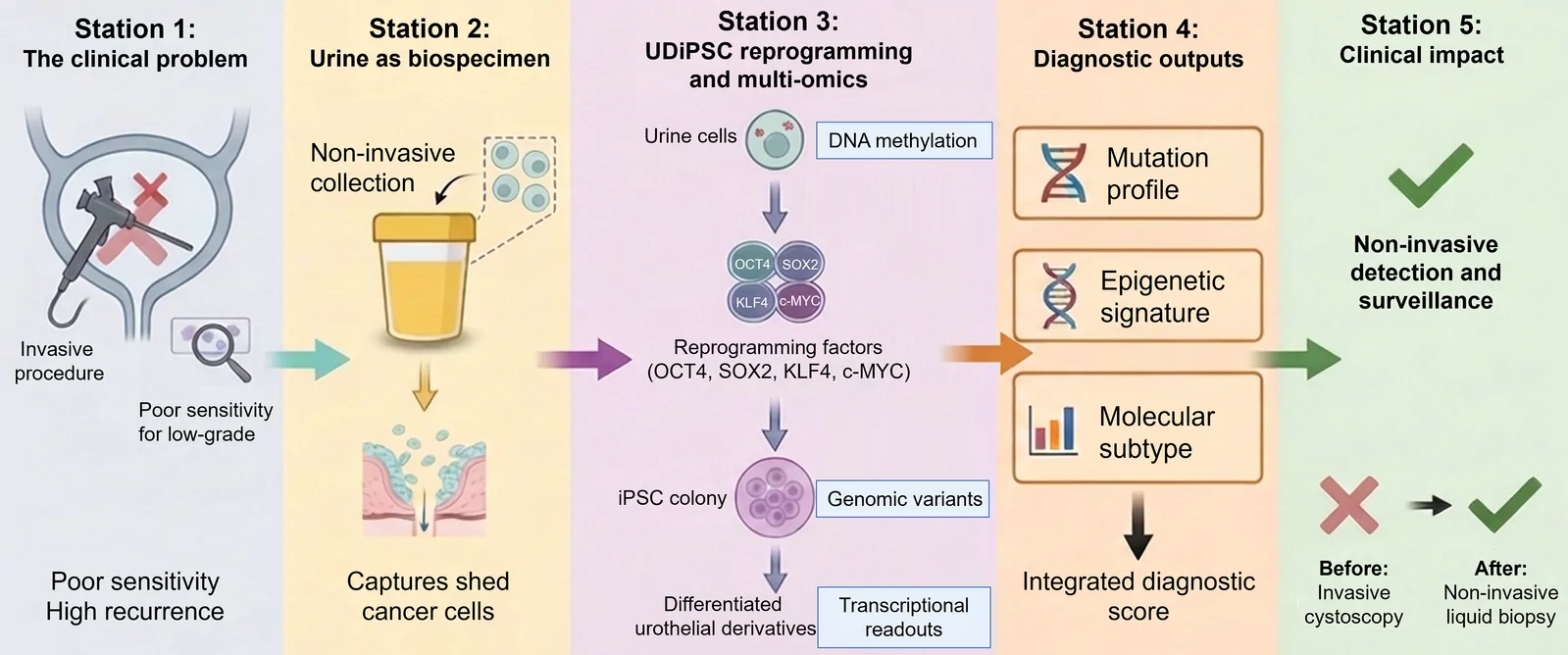
** Conceptual overview of the urine-derived induced pluripotent stem cell (UDiPSC) framework for non-invasive bladder cancer diagnostics.** Bladder cancer surveillance currently relies on invasive cystoscopy and on cytology with limited sensitivity for low-grade disease (Station 1). Voided urine offers a non-invasive, renewable biospecimen that captures exfoliated urothelial and renal epithelial cells (Station 2). DNA methylation profiling is performed on the pre-reprogramming urinary-cell fraction, where somatic methylation marks remain intact. Then, these cells can be reprogrammed via OCT4/SOX2/KLF4/c-MYC into UDiPSC clones. Reprogrammed clones enter a genomic variant-detection arm (Station 3), which generates a tiered pipeline for non-invasive bladder cancer diagnostics encompassing genomic variants from UDiPSC clones, methylation signatures obtained from the parental urinary-cell fraction, and transcriptional readouts from differentiated urothelial derivatives (Station 4). Collectively, the framework outlines a potential transition from invasive cystoscopy toward a non-invasive, cell-based liquid biopsy paradigm for detection and surveillance (Station 5). The schematic is conceptual; clinical utility remains to be established through prospective validation for bladder cancer detection and surveillance.

**Figure 2:**
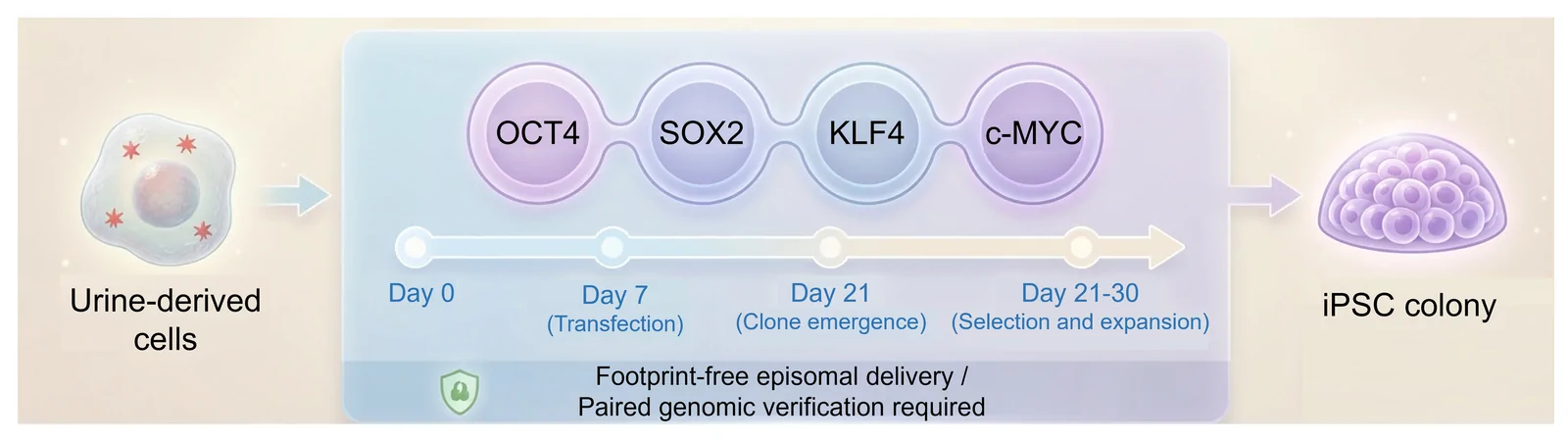
** Schematic overview of the urine-derived cell reprogramming workflow.** Non-integrating episomal reprogramming of urine-derived cells can be performed under feeder-free conditions, with colony formation typically observed within approximately 3-4 weeks 19, 20. Although this footprint-free strategy is advantageous for downstream genomic analysis, the retention of parental somatic mutations and copy-number states should be verified empirically by paired analysis of parental cells and derived iPSC clones for each intended diagnostic application.

**Figure 3:**
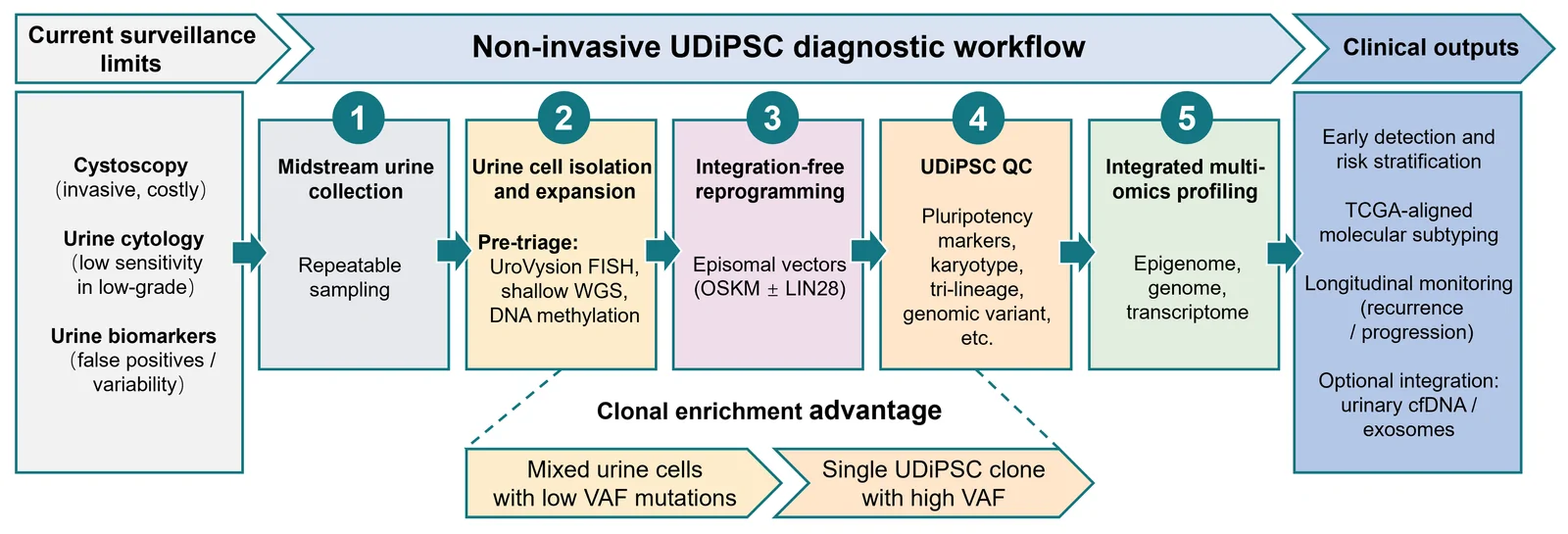
** The clonal enrichment conceptual basis of UDiPSC-based genomics and the integrated multi-omics diagnostic workflow for non-invasive BLCA detection.** In heterogeneous bulk urine-cell populations, bladder-cancer-associated alterations such as FGFR3 mutations and TERT promoter mutations may occur at low variant allele fraction (VAF) and be difficult to detect reliably by direct bulk analysis 37, 38, 40. If a mutation-carrying urine-derived cell is successfully reprogrammed, the resulting iPSC clone may reduce dilution by mutation-negative cells present in the original specimen 11, 35. This could improve detectability, although the observed VAF in any given clone will still depend on zygosity and local copy-number context 11, 28. Potential downstream outputs include identification of candidate driver alterations, exploratory comparison with established molecular frameworks in disease-relevant differentiated models, and development of integrated models for future validation. Optional integration with urinary cfDNA profiling and urinary extracellular vesicle RNA/miRNA assays represents a plausible future multianalyte extension.

**Table 1:** Comparison of candidate somatic cell sources for iPSC-based BLCA-oriented applications.

Feature	Urine-Derived Cells	Skin Fibroblasts	PBMCs	Oral Mucosa	Ref.
Collection invasiveness	Non-invasive	Minor surgical biopsy	Venipuncture	Minor biopsy or brushing	15, 23
Anatomical relevance to BLCA	Highest among non-invasive sources; may include urinary tract-derived cells, including urothelial and renal epithelial populations	No direct urothelial relevance	No direct urothelial relevance	No direct urothelial relevance	16, 17, 23
Longitudinal re-sampling	Readily repeatable	Relatively impractical	Feasible	Possible, but less practical than urine or blood	15, 23, 24
Potential access to urothelial field effects	Potentially accessible	Not directly informative	Not directly informative	Not directly informative	21, 22
Suitability for surveillance-oriented sampling	High	Low	Moderate	Low to moderate	3, 24
Practical considerations for downstream workflows	Simple collection; variable cell yield and composition	Established workflows but invasive procurement	Established collection and banking workflows	Feasible collection, but less standardized in iPSC studies	13, 23

**Table note:** Comparative features are synthesized from published studies of urine-derived cells, fibroblasts, peripheral blood mononuclear cells, and oral mucosal cells used for iPSC generation, together with literature on bladder field cancerization, surveillance acceptability, and practical workflow considerations. These comparisons are intended as qualitative summaries rather than formal head-to-head performance estimates. The reprogramming studies cited here largely utilized bulk urine epithelial cells rather than purified USCs.

**Table 2:** Reported characteristics of established diagnostic modalities and selected non-invasive tests, together with the current evidence status of UDiPSC-based testing.

Diagnostic Modality	LG Sensitivity	HG Sensitivity	Specificity	Non-Invasive	Principal Limitations	Ref.
White-light cystoscopy	Variable; lower for subtle flat or LG lesions	Generally high	Generally high	No	Invasive; operator-dependent; imperfect sensitivity for some lesions	61
Voided urine cytology	Limited, particularly for LG papillary lesions	Higher than for LG disease; generally strongest for HG disease and CIS	Generally high	Yes	Poor sensitivity for LG disease; interobserver variability	55, 62
UroVysion FISH	Lower for LG disease than for HG disease	Higher than for LG disease	Generally high, but variable across settings	Yes	Cost; technical expertise required; performance varies by setting	26, 27, 55
NMP22 BladderChek	Limited for LG disease; generally modest overall	Higher than for LG disease	Variable; reduced in benign inflammatory or hematuric conditions	Yes	False positives in benign conditions; heterogeneous performance	62, 85
Cxbladder family assays	Context-dependent; often reported to exceed cytology in selected hematuria-evaluation cohorts	High in reported studies	Variable across assay versions and study populations	Yes	Assay-specific performance; limited direct comparability across versions and cohorts; study heterogeneity	55, 86, 87
Urinary cfDNA profiling	Promising but not yet standardized; may be limited by low tumor fraction in early or LG disease	Higher than for LG disease in several studies	Generally high in selected studies, but heterogeneous	Yes	Pre-analytical and analytical heterogeneity; low-allele-frequency detection constraints	40
Urinary DNA methylation panels	~62-69.2% in reported cohorts; superior to cytology and FISH for LG disease	~89-100% in prospective studies	~83-100% across studies; 87.8% in the largest prospective cohort, below that of NMP22 and cytology in the same study	Yes	Requires validation across assay platforms; methylation signatures may vary by tumor subtype	44, 59, 60, 88
UDiPSC genomic profiling	Pending	Pending	Pending	Yes	No prospective validation; slow turnaround; high cost; workflow complexity	-

**Abbreviations:** LG, low-grade; HG, high-grade; CIS, carcinoma in situ; cfDNA, cell-free DNA; UDiPSC, urine-derived induced pluripotent stem cell. **Table note:** Reported characteristics of established tests are presented as qualitative literature-based summaries and should not be interpreted as directly comparable across all studies, because patient populations, clinical settings (hematuria workup vs surveillance), assay versions, analytical thresholds, and reference standards differ. This limitation is particularly relevant for multi-version commercial assays reported across heterogeneous study designs. UDiPSC-based diagnostic performance has not yet been established in prospective head-to-head studies.

## Abbreviations

AI: Artificial intelligence
ALK5: Activin receptor-like kinase 5
BCL2: B-cell lymphoma 2
BLCA: Bladder cancer (urothelial carcinoma)
c-MYC: Cellular myelocytomatosis oncogene
CDKN2A: Cyclin-dependent kinase inhibitor 2A
cfDNA: Cell-free DNA
CIS: Carcinoma in situ
CK20: Cytokeratin 20
CNV: Copy number variation
CRISPR: Clustered regularly interspaced short palindromic repeats
EGF: Epidermal growth factor
ESC: Embryonic stem cell
FGFR3: Fibroblast growth factor receptor 3
FISH: Fluorescence in situ hybridization
HG: High-grade
hiPSC: Human induced pluripotent stem cell
hPSC: Human pluripotent stem cell
iPSC: Induced pluripotent stem cell
KLF4: Krüppel-like factor 4
LG: Low-grade
LIN28: Lin-28 homolog A
MIBC: Muscle-invasive bladder cancer
MSC: Mesenchymal stem cell
NID2: Nidogen 2
NMIBC: Non-muscle-invasive bladder cancer
NMP22: Nuclear matrix protein 22
OCT4: Octamer-binding transcription factor 4
OSKM: OCT4, SOX2, KLF4, and c-MYC (Yamanaka reprogramming factors)
PBMCs: Peripheral blood mononuclear cells
RNA-seq: RNA sequencing
SNV: Single nucleotide variant
SOX2: SRY-box transcription factor 2
sWGS: Shallow whole-genome sequencing
TCGA: The Cancer Genome Atlas
TEER: Trans-epithelial electrical resistance
TERT: Telomerase reverse transcriptase
TGF-β: Transforming growth factor beta
TP53: Tumor protein 53
TWIST1: Twist family BHLH transcription factor 1
UDiPSC: Urine-derived induced pluripotent stem cell
Urinoids: Urine-derived bladder cancer organoids
USCs: Urine-derived stem cells
utDNA: Urinary tumor DNA
utMeMA: Urine tumor DNA methylation MassARRAY
VAF: Variant allele fraction
ZO-1: Zonula occludens-1

## References

[1] Comperat E, Amin MB, Cathomas R (2022). Current best practice for bladder cancer: a narrative review of diagnostics and treatments. Lancet.

[2] Yafi FA, Brimo F, Steinberg J (2015). Prospective analysis of sensitivity and specificity of urinary cytology and other urinary biomarkers for bladder cancer. Urol Oncol.

[3] Babjuk M, Burger M, Capoun O (2022). European Association of Urology Guidelines on Non-muscle-invasive Bladder Cancer (Ta, T1, and Carcinoma in Situ). Eur Urol.

[4] Mossanen M, Wang Y, Szymaniak J (2019). Evaluating the cost of surveillance for non-muscle-invasive bladder cancer: an analysis based on risk categories. World J Urol.

[5] Bray F, Laversanne M, Sung H (2024). Global cancer statistics 2022: GLOBOCAN estimates of incidence and mortality worldwide for 36 cancers in 185 countries. CA Cancer J Clin.

[6] Dyrskjot L, Hansel DE, Efstathiou JA (2023). Bladder cancer. Nat Rev Dis Primers.

[7] Takahashi K, Yamanaka S (2006). Induction of pluripotent stem cells from mouse embryonic and adult fibroblast cultures by defined factors. Cell.

[8] Yu J, Hu K, Smuga-Otto K (2009). Human induced pluripotent stem cells free of vector and transgene sequences. Science.

[9] Gore A, Li Z, Fung HL (2011). Somatic coding mutations in human induced pluripotent stem cells. Nature.

[10] Ruiz S, Gore A, Li Z (2013). Analysis of protein-coding mutations in hiPSCs and their possible role during somatic cell reprogramming. Nat Commun.

[11] Kosanke M, Osetek K, Haase A (2021). Reprogramming enriches for somatic cell clones with small-scale mutations in cancer-associated genes. Mol Ther.

[12] Papapetrou EP (2016). Patient-derived induced pluripotent stem cells in cancer research and precision oncology. Nat Med.

[13] Zhou T, Benda C, Dunzinger S (2012). Generation of human induced pluripotent stem cells from urine samples. Nat Protoc.

[14] Lee JH, Salci KR, Reid JC (2017). Brief Report: Human Acute Myeloid Leukemia Reprogramming to Pluripotency Is a Rare Event and Selects for Patient Hematopoietic Cells Devoid of Leukemic Mutations. Stem Cells.

[15] Zhou T, Benda C, Duzinger S (2011). Generation of induced pluripotent stem cells from urine. J Am Soc Nephrol.

[16] Zhang Y, McNeill E, Tian H (2008). Urine derived cells are a potential source for urological tissue reconstruction. J Urol.

[17] Bharadwaj S, Liu G, Shi Y (2011). Characterization of urine-derived stem cells obtained from upper urinary tract for use in cell-based urological tissue engineering. Tissue Eng Part A.

[18] Kim K, Gil M, Dayem AA (2020). Improved Isolation and Culture of Urine-Derived Stem Cells (USCs) and Enhanced Production of Immune Cells from the USC-Derived Induced Pluripotent Stem Cells. J Clin Med.

[19] Li D, Wang L, Hou J (2016). Optimized Approaches for Generation of Integration-free iPSCs from Human Urine-Derived Cells with Small Molecules and Autologous Feeder. Stem Cell Reports.

[20] Xue Y, Cai X, Wang L (2013). Generating a non-integrating human induced pluripotent stem cell bank from urine-derived cells. PLoS One.

[21] Hafner C, Knuechel R, Stoehr R (2002). Clonality of multifocal urothelial carcinomas: 10 years of molecular genetic studies. Int J Cancer.

[22] Hayashi Y, Fujita K, Nojima S (2020). TERT C228T mutation in non-malignant bladder urothelium is associated with intravesical recurrence for patients with non-muscle invasive bladder cancer. Mol Oncol.

[23] Raab S, Klingenstein M, Liebau S (2014). A Comparative View on Human Somatic Cell Sources for iPSC Generation. Stem Cells Int.

[24] Yossepowitch O, Herr HW, Donat SM (2007). Use of urinary biomarkers for bladder cancer surveillance: patient perspectives. J Urol.

[25] van den Brink SC, Sage F, Vertesy A (2017). Single-cell sequencing reveals dissociation-induced gene expression in tissue subpopulations. Nat Methods.

[26] Halling KC, Kipp BR (2008). Bladder cancer detection using FISH (UroVysion assay). Adv Anat Pathol.

[27] Hajdinjak T (2008). UroVysion FISH test for detecting urothelial cancers: meta-analysis of diagnostic accuracy and comparison with urinary cytology testing. Urol Oncol.

[28] Birkenkamp-Demtroder K, Nordentoft I, Christensen E (2016). Genomic Alterations in Liquid Biopsies from Patients with Bladder Cancer. Eur Urol.

[29] Gaignerie A, Lefort N, Rousselle M (2018). Urine-derived cells provide a readily accessible cell type for feeder-free mRNA reprogramming. Sci Rep.

[30] Warren L, Manos PD, Ahfeldt T (2010). Highly efficient reprogramming to pluripotency and directed differentiation of human cells with synthetic modified mRNA. Cell Stem Cell.

[31] Kim D, Kim CH, Moon JI (2009). Generation of human induced pluripotent stem cells by direct delivery of reprogramming proteins. Cell Stem Cell.

[32] Ludwig TE, Andrews PW, Barbaric I (2023). ISSCR standards for the use of human stem cells in basic research. Stem Cell Reports.

[33] Steichen C, Hannoun Z, Luce E (2019). Genomic integrity of human induced pluripotent stem cells: Reprogramming, differentiation and applications. World J Stem Cells.

[34] Kanchan K, Iyer K, Yanek LR (2020). Genomic integrity of human induced pluripotent stem cells across nine studies in the NHLBI NextGen program. Stem Cell Res.

[35] Young MA, Larson DE, Sun CW (2012). Background mutations in parental cells account for most of the genetic heterogeneity of induced pluripotent stem cells. Cell Stem Cell.

[36] D'Antonio M, Benaglio P, Jakubosky D (2018). Insights into the Mutational Burden of Human Induced Pluripotent Stem Cells from an Integrative Multi-Omics Approach. Cell Rep.

[37] Zuiverloon TC, van der Aa MN, van der Kwast TH (2010). Fibroblast growth factor receptor 3 mutation analysis on voided urine for surveillance of patients with low-grade non-muscle-invasive bladder cancer. Clin Cancer Res.

[38] Rachakonda PS, Hosen I, de Verdier PJ (2013). TERT promoter mutations in bladder cancer affect patient survival and disease recurrence through modification by a common polymorphism. Proc Natl Acad Sci U S A.

[39] Robertson AG, Kim J, Al-Ahmadie H (2017). Comprehensive Molecular Characterization of Muscle-Invasive Bladder Cancer. Cell.

[40] Huang FF, Di XF, Bai MH (2024). Analysis of urine cell-free DNA in bladder cancer diagnosis by emerging bioactive technologies and materials. Front Bioeng Biotechnol.

[41] Lee DS, Shin JY, Tonge PD (2014). An epigenomic roadmap to induced pluripotency reveals DNA methylation as a reprogramming modulator. Nat Commun.

[42] Renard I, Joniau S, van Cleynenbreugel B (2010). Identification and validation of the methylated TWIST1 and NID2 genes through real-time methylation-specific polymerase chain reaction assays for the noninvasive detection of primary bladder cancer in urine samples. Eur Urol.

[43] Scher MB, Elbaum MB, Mogilevkin Y (2012). Detecting DNA methylation of the BCL2, CDKN2A and NID2 genes in urine using a nested methylation specific polymerase chain reaction assay to predict bladder cancer. J Urol.

[44] Xiao Y, Ju L, Qian K (2022). Non-invasive diagnosis and surveillance of bladder cancer with driver and passenger DNA methylation in a prospective cohort study. Clin Transl Med.

[45] Kim K, Doi A, Wen B (2010). Epigenetic memory in induced pluripotent stem cells. Nature.

[46] Cancer Genome Atlas Research N (2014). Comprehensive molecular characterization of urothelial bladder carcinoma. Nature.

[47] Kamoun A, de Reynies A, Allory Y (2020). A Consensus Molecular Classification of Muscle-invasive Bladder Cancer. Eur Urol.

[48] Loriot Y, Necchi A, Park SH (2019). Erdafitinib in Locally Advanced or Metastatic Urothelial Carcinoma. N Engl J Med.

[49] Kang M, Kim HH, Han YM (2014). Generation of bladder urothelium from human pluripotent stem cells under chemically defined serum- and feeder-free system. Int J Mol Sci.

[50] Suzuki K, Koyanagi-Aoi M, Uehara K (2019). Directed differentiation of human induced pluripotent stem cells into mature stratified bladder urothelium. Sci Rep.

[51] Lee SH, Hu W, Matulay JT (2018). Tumor Evolution and Drug Response in Patient-Derived Organoid Models of Bladder Cancer. Cell.

[52] Viergever BJ, Raats DAE, Geurts V (2024). Urine-derived bladder cancer organoids (urinoids) as a tool for cancer longitudinal response monitoring and therapy adaptation. Br J Cancer.

[53] Walz S, Pollehne P, Geng R (2023). A Protocol for Organoids from the Urine of Bladder Cancer Patients. Cells.

[54] Liu J, Zhijin Z, Zhang W (2024). Urinary exosomes: Potential diagnostic markers and application in bladder cancer. Heliyon.

[55] Heard JR, Mitra AP (2024). Noninvasive Tests for Bladder Cancer Detection and Surveillance: A Systematic Review of Commercially Available Assays. Bladder Cancer.

[56] Lin H, Shi X, Li H (2021). Urinary Exosomal miRNAs as biomarkers of bladder Cancer and experimental verification of mechanism of miR-93-5p in bladder Cancer. BMC Cancer.

[57] Huang H, Du J, Jin B (2021). Combination of Urine Exosomal mRNAs and lncRNAs as Novel Diagnostic Biomarkers for Bladder Cancer. Front Oncol.

[58] Cohen JD, Li L, Wang Y (2018). Detection and localization of surgically resectable cancers with a multi-analyte blood test. Science.

[59] Chen X, Zhang J, Ruan W (2020). Urine DNA methylation assay enables early detection and recurrence monitoring for bladder cancer. J Clin Invest.

[60] Jeong IG, Yun SC, Ha HK (2025). Urinary DNA Methylation Test for Bladder Cancer Diagnosis. JAMA Oncol.

[61] Devlies W, de Jong JJ, Hofmann F (2024). The Diagnostic Accuracy of Cystoscopy for Detecting Bladder Cancer in Adults Presenting with Haematuria: A Systematic Review from the European Association of Urology Guidelines Office. Eur Urol Focus.

[62] Chou R, Gore JL, Buckley D (2015). Urinary Biomarkers for Diagnosis of Bladder Cancer: A Systematic Review and Meta-analysis. Ann Intern Med.

[63] Huangfu D, Maehr R, Guo W (2008). Induction of pluripotent stem cells by defined factors is greatly improved by small-molecule compounds. Nat Biotechnol.

[64] Lin T, Ambasudhan R, Yuan X (2009). A chemical platform for improved induction of human iPSCs. Nat Methods.

[65] Jordaens S, Zwaenepoel K, Tjalma W (2023). Urine biomarkers in cancer detection: A systematic review of preanalytical parameters and applied methods. Int J Cancer.

[66] Jha BS, Farnoodian M, Bharti K (2021). Regulatory considerations for developing a phase I investigational new drug application for autologous induced pluripotent stem cells-based therapy product. Stem Cells Transl Med.

[67] Novoa J, Westra I, Steeneveld E (2024). Validating human induced pluripotent stem cell-specific quality control tests for the release of an intermediate drug product in a Good Manufacturing Practice quality system. Cytotherapy.

[68] Li MM, Datto M, Duncavage EJ (2017). Standards and Guidelines for the Interpretation and Reporting of Sequence Variants in Cancer: A Joint Consensus Recommendation of the Association for Molecular Pathology, American Society of Clinical Oncology, and College of American Pathologists. J Mol Diagn.

[69] Song SJ, Nam Y, Rim YA (2024). Comparative analysis of regulations and studies on stem cell therapies: focusing on induced pluripotent stem cell (iPSC)-based treatments. Stem Cell Res Ther.

[70] Bohrer LR, Stone NE, Mullin NK (2023). Automating iPSC generation to enable autologous photoreceptor cell replacement therapy. J Transl Med.

[71] Gordon LG, White NM, Elliott TM (2020). Estimating the costs of genomic sequencing in cancer control. BMC Health Serv Res.

[72] Clark O, Sarmento T, Eccleston A (2024). Economic Impact of Bladder Cancer in the USA. Pharmacoecon Open.

[73] Yeung C, Dinh T, Lee J (2014). The health economics of bladder cancer: an updated review of the published literature. Pharmacoeconomics.

[74] Zhang X, Wang J, Lu J (2021). Robust Prognostic Subtyping of Muscle-Invasive Bladder Cancer Revealed by Deep Learning-Based Multi-Omics Data Integration. Front Oncol.

[75] Wang X, Wu C, Zhang S (2022). A novel deep learning segmentation model for organoid-based drug screening. Front Pharmacol.

[76] Reyes M, Meier R, Pereira S (2020). On the Interpretability of Artificial Intelligence in Radiology: Challenges and Opportunities. Radiol Artif Intell.

[77] Sarma KV, Harmon S, Sanford T (2021). Federated learning improves site performance in multicenter deep learning without data sharing. J Am Med Inform Assoc.

[78] Ungricht R, Guibbal L, Lasbennes MC (2022). Genome-wide screening in human kidney organoids identifies developmental and disease-related aspects of nephrogenesis. Cell Stem Cell.

[79] Ringel T, Frey N, Ringnalda F (2020). Genome-Scale CRISPR Screening in Human Intestinal Organoids Identifies Drivers of TGF-beta Resistance. Cell Stem Cell.

[80] Michels BE, Mosa MH, Streibl BI (2020). Pooled In Vitro and In Vivo CRISPR-Cas9 Screening Identifies Tumor Suppressors in Human Colon Organoids. Cell Stem Cell.

[81] Zhang SZ, Ma LX, Qian WJ (2016). Modeling Neurological Disease by Rapid Conversion of Human Urine Cells into Functional Neurons. Stem Cells Int.

[82] Nagai H, Saito M, Iwata H (2024). Direct conversion of urine-derived cells into functional motor neuron-like cells by defined transcription factors. Sci Rep.

[83] Yin X, Li Q, Shu Y (2024). Exploiting urine-derived induced pluripotent stem cells for advancing precision medicine in cell therapy, disease modeling, and drug testing. J Biomed Sci.

[84] Tan WS, Feber A, Dong L (2017). DETECT I & DETECT II: a study protocol for a prospective multicentre observational study to validate the UroMark assay for the detection of bladder cancer from urinary cells. BMC Cancer.

[85] Wang Z, Que H, Suo C (2017). Evaluation of the NMP22 BladderChek test for detecting bladder cancer: a systematic review and meta-analysis. Oncotarget.

[86] O'Sullivan P, Sharples K, Dalphin M (2012). A multigene urine test for the detection and stratification of bladder cancer in patients presenting with hematuria. J Urol.

[87] Harvey JC, Cambridge LM, Ellen CW (2024). Analytical Validation of Cxbladder((R)) Detect, Triage, and Monitor: Assays for Detection and Management of Urothelial Carcinoma. Diagnostics (Basel).

[88] Green EA, Li R, Albiges L (2021). Clinical Utility of Cell-free and Circulating Tumor DNA in Kidney and Bladder Cancer: A Critical Review of Current Literature. Eur Urol Oncol.

